# Thermo-Sensitive Vesicles in Controlled Drug Delivery for Chemotherapy

**DOI:** 10.3390/pharmaceutics10030150

**Published:** 2018-09-05

**Authors:** Elisabetta Mazzotta, Lorena Tavano, Rita Muzzalupo

**Affiliations:** Department of Pharmacy, Health and Nutritional Sciences, University of Calabria, Via Savinio, Ed. Polifunzionale, 87036 Arcavacata di Rende, Italy; mazzotta-elisabetta@libero.it (E.M.); uclorena@tiscali.it (L.T.)

**Keywords:** thermo-sensitivity, hyperthermia, liposomes, vesicles, drug delivery, cancer

## Abstract

Thermo-sensitive vesicles are a promising tool for triggering the release of drugs to solid tumours when used in combination with mild hyperthermia. Responsivity to temperature makes them intelligent nanodevices able to provide a site-specific chemotherapy. Following a brief introduction concerning hyperthermia and its advantageous combination with vesicular systems, recent investigations on thermo-sensitive vesicles useful for controlled drug delivery in cancer treatment are reported in this review. In particular, the influence of bilayer composition on the in vitro and in vivo behaviour of thermo-sensitive formulations currently under investigation have been extensively explored.

## 1. Introduction to Novel Approaches for Chemotherapy

The treatment of cancer with antineoplastic drugs is known as chemotherapy [1]. Generally, chemotherapeutics are administered intravenously and, due to their systemic distribution, the therapeutic effect at the tumour site is achieved only after high dose administration, often causing resistance and severe adverse effects [2,3].

In recent decades, drug delivery systems (DDS) have been designated as the solution to systemic toxicity due to chemotherapy. DDS are versatile systems since they can be designed in order to encapsulate the opportune amount of drug that reaches the tumour site and releases a sufficient amount of chemotherapeutics to produce an effective therapeutic response [4]. Among several macromolecular carriers, vesicular systems such as liposomes and niosomes, have been widely investigated and proposed as the most promising [5]. First, vesicles were only designed to provide site-specific treatment, while the second-generation of such systems also possesses the ability to trigger drug release, assuring higher control of the therapy. Among stimuli-sensitive approaches, temperature-sensitive vesicles (TSV) have been successfully developed, with the commercial ThermoDox^®^ version currently undergoing phase III clinical trials. After being administered intravenously, ThermoDox^®^ (TSL-Dox) in combination with radiofrequency ablation (RFA) leverages two typical features of tumour biology to deliver higher concentrations of drug directly to the tumour site. First, the rapidly growing tumours have a leaky vasculature and, therefore, are permeable to liposomes, enabling their accumulation within tumours. This mechanism is known as the enhanced permeability and retention (EPR) effect. Second, when the tumour tissue is heated at about 40 °C, the thermosensitive carriers rapidly change their structure and the selectively destabilized liposomal membrane releases the drug directly into the tumour and the surrounding vasculature [6]. Therefore, systemic delivery of an anticancer drug loaded into thermo-sensitive vesicles represents a strategy that allows both the local control of release using mild hyperthermia and the subsequent accumulation of the drug by diffusion in the tumour mass, ensuring minimal exposure to the drug in normal tissue [7]. There are several clinical trials with TSL-Dox, all with the formulation ThermoDox^®^ in combination with various heating modalities. In human clinical trials, as heating sources, high-intensity-focused ultrasound (HIFU) in combination with magnetic resonance imaging (MRgHIFU), and for animal clinical trials microwave devices and various light sources, are used [8].

Recent investigations on thermo-sensitive vesicles useful for controlled drug delivery in cancer treatment, focusing on the influence of bilayer composition on the in vitro and in vivo behaviour of thermo-sensitive formulations, are reported here.

## 2. Hyperthermia and Temperature-Sensitive Vesicles (TSV)

### 2.1. Hyperthermia and Cancer: General Remarks

**Figure 1:**
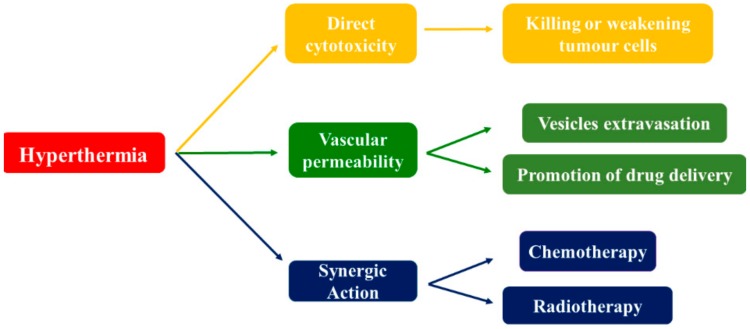
Hyperthermia most important therapeutic benefits.

Hyperthermia is defined as the procedure of raising the temperature of the tumour tissue to 40–43 °C, and it has been used in the treatment of several diseases, including cancer, showing many therapeutic benefits, as illustrated in Figure 1 [9].

The most obvious advantage of hyperthermia is its direct cytotoxicity: it may kill or damage tumour cells, with limited effects on healthy cells. This is due to the disorganized and compact vascular structure of cancer cells, which prevents heat dissipation and, in combination with tumour oxygenation, can induce a cell final necrotic or apoptotic death [10,11]. When cells in the body are exposed to temperatures higher than normal (i.e., 42 °C), several changes occur, making them more sensitive to other treatments (such as chemotherapy and radiotherapy), due to synergistic action resulting in an enhanced cytotoxic effect [12]. Accordingly, hyperthermia is mainly used as an adjuvant therapy [13]. Additionally, hyperthermia treatment is claimed to improve tumour blood flow and vascular permeability and to increase vessels pore size. Therefore, an intensified vesicle extravasation nearby the tumour site, improving drug delivery, has been demonstrated [14].

Obviously, killing the cells depends both on the length of treatment and temperature achieved during the therapeutic sessions and it has been reported that thermo-tolerance, consisting in a transient resistance to additional heat stress, occurs when hyperthermia sessions have been given in an interval shorter than 48–72 h [15]. For these reasons, hyperthermia must be carefully controlled and monitored. The higher the temperature and the longer the time that heat is applied to the tumour, the stronger is the lethal effect induced [15].

According to the extension of the treatment area, hyperthermia can be local, regional or whole-body, giving heat delivery to localized, advanced or deep and widespread cancer, respectively [16]. Generally, a number of techniques including ultrasound, microwaves, radiofrequency, ferromagnetic seeds and resistive wire implants have been used to heat the tumour site. Local hyperthermia is used for small tumours located superficially or within an accessible body cavity (i.e., rectum or esophagus), while regional hyperthermia is often destined to treat advanced tumours affecting large parts of the body, organs or limbs (major and minor pelvis, abdomen or thighs). Widespread cancerous cells may be destroyed or sensitized to drugs by achieving a systemic temperature of 42.0 °C in the whole organism: this mechanism is called whole-body hyperthermia and can be administered only after deep analgesia and sedation or general anesthesia [17].

### 2.2. Hyperthermia and Its Combination with Vesicular Systems

Vesicular systems able to release drugs after heating of few degrees above physiological temperature represent an attractive strategy to treat cancer, due to the possibility to control drug release by changing the heating focus and power [18]. The first temperature-sensitive formulation of such systems was designed in 1978 by Yatvin et al. [19], and since then, a lot of smart thermo-sensitive nanodevices have been proposed [20], with the first formulation demonstrating its in vivo efficacy in 2000 [7] and entering human clinical trials in 2011 as ThermoDox^®^ [6].

In recent decades, additional and combined approaches to improve the performance of classical thermo-sensitive vesicles have been reported. For instance, a pre-hyperthermia treatment has been proposed to improve tumour vasculature permeability for passive carrier accumulation, followed by a second heat trigger producing interstitial drug release [21]. Additionally, surface modifications for active targeting of the tumour vasculature or tumour cells have been proposed [22]. 

### 2.3. Ideal Thermo-Sensitive Vesicles

**Figure 2:**
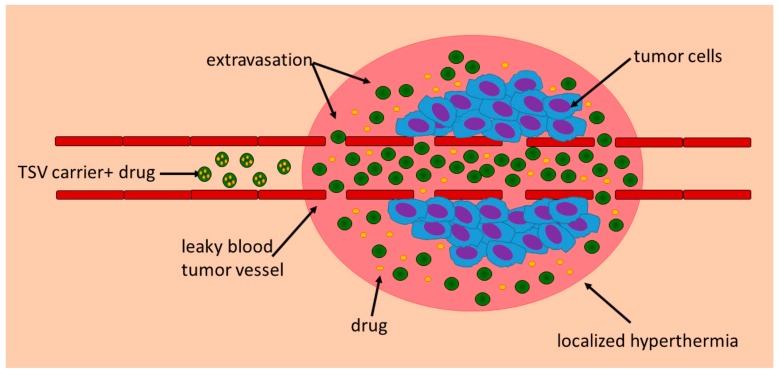
Schematic mechanisms involved in combining hyperthermia and thermo-sensitive devices therapy.

An ideal thermo-responsive nanodevice should be obtained by materials that are safe and sensitive enough to respond to temperature changes between 39 °C and 42 °C. Furthermore, the device may be able to sequester a drug until it reaches the tumour site, where hyperthermia can promote carrier extravasation and a localized triggered release, ensuring minimal drug exposure for normal tissue (Figure 2) [14].

**Figure 3:**
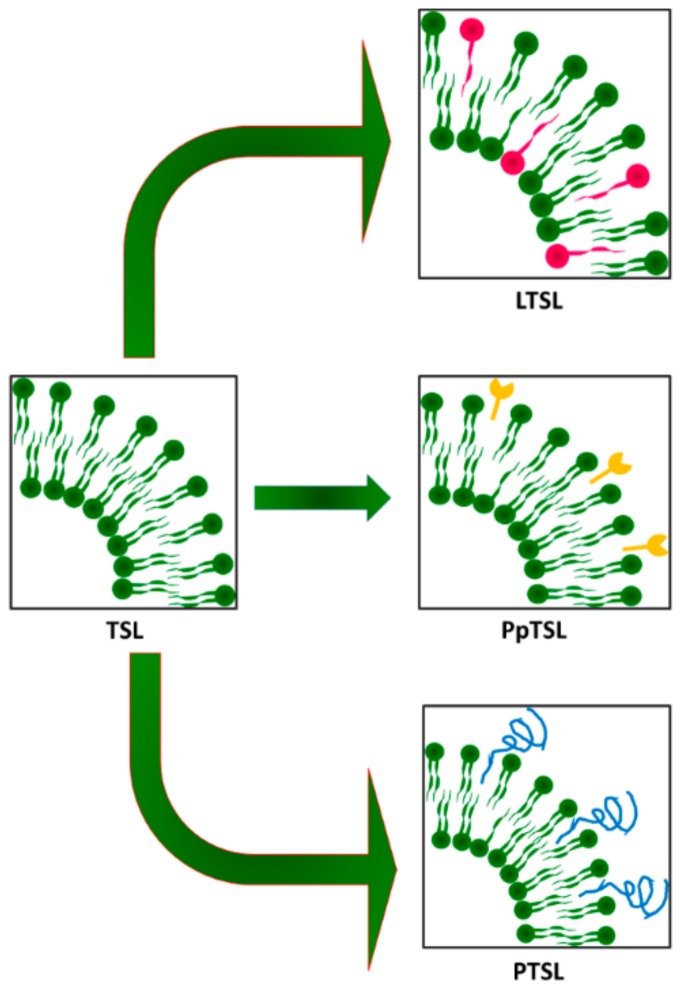
Schematic representation of different thermo-sensitive liposome (TSL) systems: lysolipids thermo-sensitive liposomes (LTSL), peptides thermo-sensitive liposomes (PpTSL), polymer thermo-sensitive liposomes (PTSL).

Traditional thermo-sensitive vesicles are composed of lipids or non-ionic surfactants that undergo a gel-to-liquid phase transition slightly above 37 °C, whereas more recently, temperature-sensitization of nanosized carriers has been demonstrated with the use of lysolipids and synthetic temperature-sensitive polymers, as illustrated in Figure 3 and extensively explored in the following section [18].

**Figure 4:**
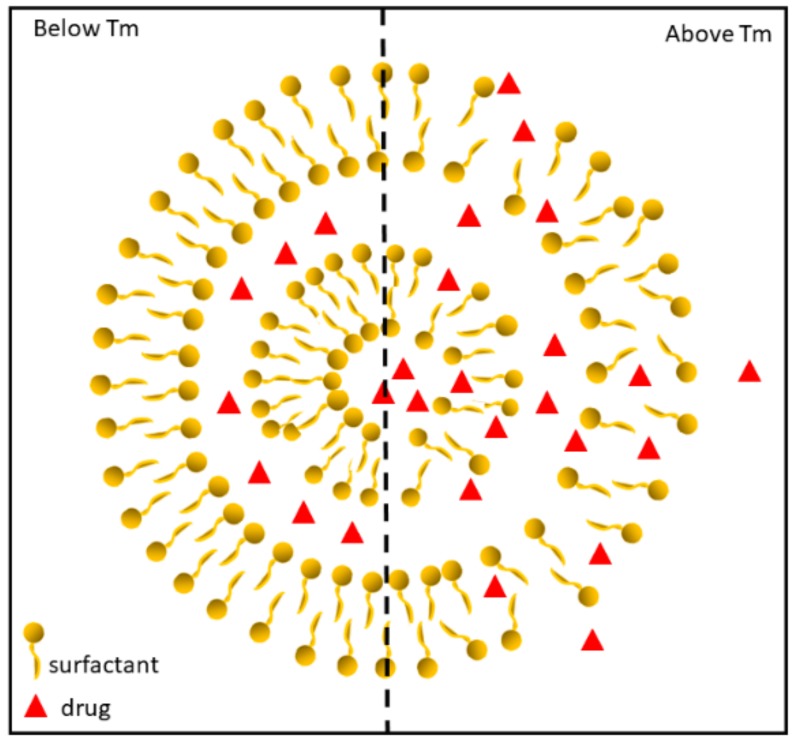
Phase transition behaviour of temperature-sensitive vesicles (TSV). Below *T*_m_, lipid membranes exist in solid phase and therefore no drug release is expected. When the temperature of lipid membrane passes through *T*_m_, the bilayer permeability increases, promoting drug release.

The most important feature of a phospholipidic or non-ionic surfactant membrane is the existence of a temperature-dependent reversible phase transition (*T*_m_), in which the phospholipid or surfactant hydrocarbon chains undergo a transition from an ordered (gel) to a more disordered fluid (liquid crystalline) state. In the gel phase, lipid molecules are ordered and condensed with fully extended hydrocarbon chains, and are constrained to the two-dimensional plane of the membrane. Upon heating, the mobility of the lipid head groups gradually increases. As the temperature is further increased and is close to *T*_m_, the orientation of the C–C single bonds in the hydrocarbon chains begins to switch from a *trans* to a *gauche* configuration. At temperatures higher than *T*_m_, the bilayer exists in a fully liquid phase. Individual lipid molecules are still confined to the two-dimensional plane of the membrane as in the solid phase, but they are able to move freely and rapidly within the plane, as illustrated in Figure 4 [23]. Obviously, the physical state of the bilayer affects the permeability, leakage, and overall stability of the liposomes and the *T*_m_ is a function of the lipid mixture, whereby it can be altered by changing the bilayer composition [24].

## 3. Recent Advances on Thermo-Sensitive Vesicles for Controlled Drug Delivery

Since the first temperature-sensitive formulation in 1978, several approaches were further developed in order to optimize carrier performances. Below, we report a detailed discussion on the most representative thermo-sensitive vesicular systems, which are classified and commented on according to the strategy for which why they were designed, and focus on the potential of their peculiarities.

### 3.1. Traditional Thermo-Sensitive Liposomes

To ensure the therapeutic success of TSL, the *T*_m_ must be preferably a few degrees above the physiological temperature and in the range of typical temperatures of the mild hyperthermia. 

1,2-dypalmitoyl-*sn*-glycero-3-phosphocoline (DPPC) which has a *T*_m_ of 41.4 °C is used as a major component in most TSL formulations, despite this phospholipid has been reported to limit drug release [19,25].

The first thermo-sensitive liposome (TSL), introduced by Yatvin and collaborators in 1978 was made of DPPC and 1,2-distearoyl-*sn*-glycero-3-phosphocholine (DSPC) in 3:1 molar ratio. The *T*_m_ obtained depended on the lipid combination and it ranged between 42.5 and 44.5 °C, and in this temperature interval drug release occurred [19].

After the pioneering study of Yatvin [19], a lot of TSL formulations based on mixtures of DPPC and other phospholipids, have been designed and studied, with the objective to improve in vitro vesicles stability, to increase the drug release rate [26] and demonstrating that the combination of TSL and hyperthermia is really able to increase the level of several cytotoxic drugs at the tumour site and enhance their therapeutic effect [27,28]. 

As reported in Table 1, the inclusion of other components such as cholesterol (CH) and modified-polyethylene glycol (PEGs) into the lipid bilayer, can prolong the liposome circulation time together with a significant enhancement in their content release upon heating. CH was introduced to optimize vesicle stability in serum [26]. The presence of CH in the bilayer is able to reduce the undesired drug leakage at 37 °C, when the temperature is below the *T*_m_ [29]. Another issue of TSL obtained by using conventional phospholipids is the rapid elimination by the reticuloendothelial systems (RES), that limits the bioavailability of the vehiculated drug. To overcome this drawback, the design of stealth liposomes by using PEG-lipids has been proposed [30].

Interesting results have been obtained by Dicheva and collaborators by the introduction for the first time of cationic lipids in the bilayer composition. Indeed, cationic nanocarriers have been reported to deliver selectively anticancer drugs to angiogenic endothelial cells and tumour cells. This strategy provides an important targeting to endothelial and tumour cells compared to non-cationic formulations [45,46,47].

Other studies have been carried out by preparing TSL with a multifunctional target or loaded with different bioactive molecules. An interesting multi-functional approach was proposed by Pradhan in 2010. The researchers described folate receptor targeted thermo-sensitive magnetic liposomes, and showed that this formulation that integrate active targeting, magnetic field gradient targeting, drug temperature triggered release and pharmacological activity can be used advantageously for thermo-chemotherapy of cancers [31]. Traditional TSL have been also proposed for drug co-encapsulation, as reported by de Smet and collaborators in 2013, combining doxorubicin and gadolinium (GD) in ^111^In-labeled liposomes. In this study, the authors used high-intensity focused ultrasound (HIFU) to obtain deep and local hyperthermia combined with magnetic resonance imaging (MRI) to allow cancer diagnosis and treatment. The result has shown that HIFU-mediated hyperthermia of the tumour increased more than a 4-fold higher uptake of the radiolabeled TSL while the doxorubicin concentration increased about 8-fold in the tumour [32].

Another study that investigated the simultaneous release of Gemcitabine (Gem) and GD was realized by Affram et al. [48]. In this study the researchers prepared two TSL delivery systems with one encapsulated with Gem, a poor membrane-permeable drug, and another encapsulated only with GD. They demonstrated that this strategy improved the antitumour efficacy of Gem, and increased distribution of Gem and GD in tissues and organs.

In addition to bilayer composition, the in vitro and in vivo drug release rate from TSL is influenced by many factors such as physico-chemical properties of the carriers (i.e., vesicles size), and chemical and biological characteristics of the environment in which they are located after the injection (i.e., the presence of serum and its composition). An in–depth study devoted to the evaluation of the vesicles size influence on drug release after intravenous application was proposed in 2010 by Hossann and collaborators [49] and they concluded that in vitro release properties of TSL are dramatically influenced by vesicle size in the range of 50 to 200 nm. With decreasing vesicle size the content release rates is increased due to the membrane curvature increasing, resulting in more packing defects and consequently more membrane permeability. Furthermore, TSL stability at 37 °C in serum is also dramatically affected and several studies demonstrated how even the drug release is influenced by the presence of serum. In fact, as reported in literature, the components possess a destabilizing effect on the bilayer of vesicles, because the interaction of serum components with the membrane bilayer appears to increase the diameter of packing defects or pores, followed by an increase in release rates of hydrophilic compounds [50]. The research group of Hossann in 2012 evaluated the effect of two major serum proteins, albumin (HSA) and immunoglobulin type G (IgG), on the stability and integrity of the following TSL formulations: DPPC/DSPC/DPPG_2_(DPPG_2_-TSL), DPPC/DSPC/DPPG_2_/DSPE-PEG_2000_ (DPPG_2_/PEG-TSL), DPPC/P-Lyso-PC/DSPE-PEG_2000_ (PEG/Lyso-TSL), and DPPC/DSPC/DSPE-PEG_2000_ (PEG-TSL). In particular, HSA was reported to increase the carboxyfluorescein (CF) release from all formulations around *T*_m_, while IgG was found to affect only anionic TSL. Moreover, the CF release increased around or above the *T*_m_, dependently on the HSA concentration, but below the *T*_m_ the protein stabilized the bilayer [51].

Unfortunately, the purported low stability of TSL in blood circulation limited also their capacity to reach the site action in effective doses, because of their reduced plasma half-life. A high stability in serum, associated to the enhanced permeability and retention (EPR) effect, may allow the drug to accumulate at the desired site in concentrations high enough to produce a therapeutic effect. With the aim of improving TSL-mediated drug delivery, the surface of liposomes is often modified with a hydrophilic polymer such as PEG that, as reported before, is able to provide steric protection to the carrier by increasing its surface hydrophilicity and consequently reducing the binding of serum component, preventing opsonisation and limiting its capture by the RES [52]. An exhaustive study on the use of DSPE-PEG_2000_ has been reported by Li and collaborators by incorporating this compound into the bilayer and evaluating the optimal concentration of grafted polymer on the liposomal surface to stabilize the vesicles in serum and to improve the release efficiency under mild hyperthermia. The results suggested that the incorporation of 5% mol DSPE-PEG_2000_ is sufficient to stabilize the lipid membrane in serum at physiological temperature and to enhance the kinetics release at 42 °C. Moreover, the authors demonstrated that the use of higher density DSPE-PEG_2000_ may cause the membrane integrity collapse, producing a significant CF release [53].

The main approaches developed to overcome the limited drug release occurring in traditional TSL include the incorporation of thermo-sensitive polymer or lysolipids into the bilayer, or a change in the methodology treatment [18]. Recently, Li and collaborators have proposed a novel two step mild hyperthermia approach to further improve the therapeutic performances of DPPC/DSPC/DSPE-PEG_2000_-based TSL [36]. The first step of mild hyperthermia (41 °C) increases tumour vasculature permeability and maximizes intratumoral liposomal drug accumulation; the second step promotes the drug release from TSL, with minimal drug redistribution through circulation. This type of heating protocol is known as the interstitial release approach. The authors observed that the liposome accumulation and DOX bioavailability at the tumour site obtained with the two-step approach is increased when compared to the one-step classical treatment, resulting in being particularly beneficial for large and deep-seated tumours [36]. The effect of heating protocol on drug release profile from TSL has been also investigated by Al-Ahmady et al. The research group developed a new type of TSV by inclusion of leucine zipper peptides within a lipid bilayer (Lp-Peptide hybrids) and investigated their activity in vivo using two different heating protocols: the first was an interstitial release approach; the second was an intravascular release protocol in which TSL are administered during the heating process, resulting in drug release inside blood vessels when reaching the heated area. Both methods proved to be effective, but the suppression of tumour growth was greater with the intravascular approach. This study, therefore, highlights the importance of the choice of the heating protocol that in turn depends on the physical-chemical characteristics and on the pharmacokinetic profile of the TSV to improve clinical efficacy [54]. 

### 3.2. Influence of Lysolipids on Thermo-Sensitive Liposome (TSL) Properties

Lysolipids (LP) are phospholipids in which one or both acyl group derivatives have been removed. Their non-cylindrical structure allows them to be easily incorporated into a lipid membrane and to alter the chemical and physical properties of the bilayer, such as membrane permeability, morphology and stability. The incorporation of a small amount of LP leads to a destabilization and reduction of membrane ability to act as a barrier, due to changes in membrane curvature caused by the particular geometry of lysolipids [55]. Moreover, the presence of a lysolipid in the bilayer has been reported to reduce the phase transition temperature of traditional thermo-sensitive liposomes from 43 °C to 39–40 °C and, furthermore, their accumulation at the grain boundaries and the formation of stabilized defects may result in an increased drug release rate [56]. The decrease of *T*_m_ is necessary and clinical trials recommend mild HT <43 °C because higher temperature can cause hemorrhage or damage to the surrounding healthy tissue [6].

**Figure 5:**
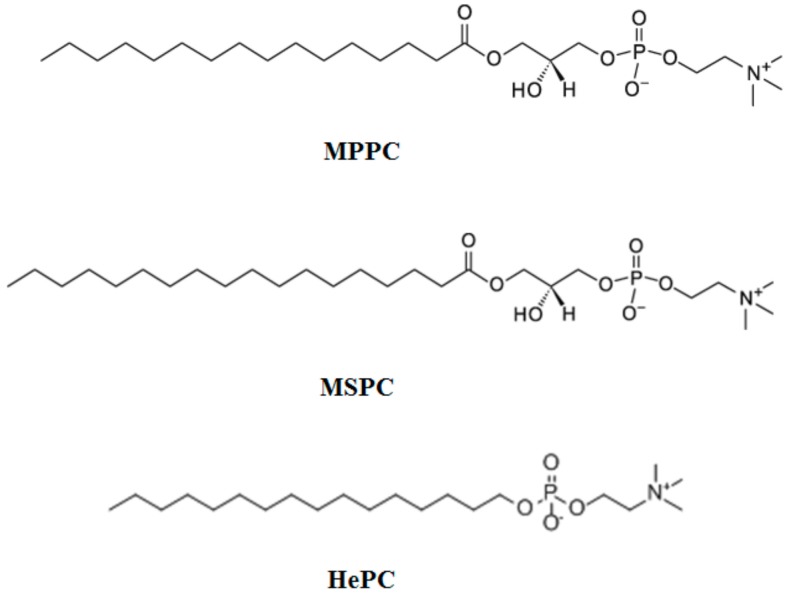
Most frequently used lysolipids.

The most frequently used lysolipids are shown in Figure 5.

The first example of lysolipids-modified TSL was proposed in 1999 by Anyarambhatla and collaborators, incorporating LP in PEGylated TSL, with the aim to decrease the *T*_m_ phase transition and to promote a rapid drug release [57]. This first formulation originally comprised DPPC/MSPC/DSPE-PEG2000 in 90:10:4 molar ratio and since then slight bilayer modifications have been proposed, reviewed as well by Landon in 2011 [6].

Typically, traditional TSL were able to release a drug over 30 min, while after the lysolipids inclusion into liposomes bilayer, drug release occurs in a matter of seconds [58]. 

In 2010, de Smet and collaborators [59] studied the simultaneous release of doxorubicin and gadolinium, a contrast agent for magnetic resonance image (MRI) guidance, co-encapsulating them in two different thermo-sensitive systems: a traditional liposomal sample made of DPPC/HSPC/CH /DPPE-PEG_2000_ and a LTSL system based on DPPC/MPPC/DPPE-PEG_2000_. Drug release from LTSL formulation was faster than that obtained from the classical TSL formulation at 42 °C, as required by an ideal thermo-sensitive system, but unlike TSL, this formulation presented also an unwanted leakage of doxorubicin at 37 °C [59]. The presence of lysolipids, in fact, determined a certain vesicle’s sensitivity to the serum, due to their ability to make the liposomal bilayer interact with HSA or exchange and incorporate themselves into cellular membranes [60]. This desorption from the bilayer led to a premature drug leakage at physiological temperature, invalidating the clinical success of low thermo-sensitive vesicles. Nevertheless, the co-encapsulation of MRI contrast agent did not influence the loading and release kinetics of DOX and this suggested that their simultaneous release makes possible the in vivo monitoring and control of the drug delivery process [60].

Different thermo-sensitive formulations encapsulating carboxyfluorescein (CF) were developed starting from mixtures of DPPC, DSPC, P-Lyso-PC, DSPE-PEG_2000_, DPPG_2_ and hexadecylphosphocholine (HePC). Results demonstrated that the CF release rate increased with decreasing liposomes size, in the temperature range between 30 and 45 °C. Probably, this is due to an increase of membrane curvature, resulting in more packing defects and consequently in a higher membrane permeability, as reported in literature [61]. Nevertheless, release rate depended on the composition of the bilayer: while CF release from DPPG_2_-TSL is strongly affected by vesicle size, a similar behaviour was not observed for DPPG_2_/HePC-TSL [34]. Furthermore, the CF release appeared also to be higher in the presence of serum, due to the highest interaction of albumin with DPPC-TSL and DPPG-TSL, resulting in an increased diameter of packing defects and release rates. 

In 2007, Hossann and collaborators proposed a novel TSL formulation with a prolonged plasma half-life without the use of PEGylated lipids, commonly used strategy, but based on DPPGOG [33]. Since the only TSL formulation in clinical trials applies DSPE-PEG2000 and lysophosphatidylcholine (P-lyso-PC), the main objective of this study was to compare the influence of DPPGOG, DSPE-PEG_2000_ and P-lyso-PC on in vitro vesicles stability and on thermal-triggered drug release. Three different formulations were developed and compared in presence of fetal calf serum: DPPC/DSPC/DPPGOG 50:20:30, DPPC/P-lyso-PC/DSPEPEG_2000_ 90:10:4 and PEGylated based TSL. The DPPGOG based formulation showed an improved stability at 37 °C compared to PEGylated liposomes. In DPPC/DSPC/DPPGOG sample, CF was retained up to 10 h at 37 °C in serum, whereas PEGylated TSL became unstable after 6 h. Furthermore, DPPGOG was reported to increase membrane permeability similarly to P-lyso-PC, releasing over the 70% of CF at their *T*_m_. All these properties were retained when CF was replaced with DOX. In fact, DPPGOG-TSL retained 89% of DOX and released it completely only at 42 °C. In conclusion, DPPGOG was able to prolong the liposomes in vivo half-life, like DSPE-PEG_2000_, and to increase the drug release like P-lyso-PC, without a negative effect on TSL stability [33].

Cisplatin-loaded LTSL were designed by Woo and collaborators in 2008, proposing a new drug encapsulation method, named passive equilibration, consisting in drug loading in preformed LTSL. The researchers demonstrated that cisplatin was released from LTSL at 42 °C within 5 min [62]. 

The potential of LTSL for high molecular weight molecule delivery was studied by Zhang and collaborators [63]. Fluorescein isothiocyanate conjugate-albumin was used as a model drug (MW 66 kDa) and incorporated into liposomes, demonstrating not only excellent stability at physiological temperature, but also a fast release behaviour at 42 and 44.5 °C [63].

In 2011, Tagami and collaborators hypothesized that Brij surfactants could replace the functions of MSPC, a single acyl chain lipid/surfactant, and DSPE-PEG2000, a PEGylated lipid/surfactant in the LTSL formulation, to obtain a simplified thermo-sensitive carrier [64]. The results showed that only Brij78, may be optimally incorporated both via the thin film method or post-insertion approach, achieving formulations with better performances compared to traditional lysolipids-based thermo-liposomes. In particular, Brij78-based formulations showed stability at 37 °C comparable to that of LTSL and a fast DOX release at 40–42 °C already within 2–3 min [64].

More recently, new thermo-sensitive vesicles based on a alkylphosphocholines, a novel class of substances with anticancer and antiprotozoal activities (structurally related to lysophospolipids), have been designed [14]. Hexadecylphosphocholine (HePC) is one of the most representative molecules and presents increased metabolic stability compared with lysolipids. Additionally, unlike classical cytostatic drugs, HePC is not myelosuppressive and can stimulate leukopoiesis and thrombopoesis [65]. HePC is able to act at the same time as drug and carrier constituents, conferring peculiar and interesting functionality to the formulation, allowing the use of additional excipients to be bypassed, increasing system biocompatibility, and making them suitable candidates for special biomedical applications [14].

In 2008, Lindner and collaborators decided to investigate the HePC ability to induce a burst release from DPPGOG-based TSL by mild hyperthermia. As expected, HePC increased the CF release rate similarly to lysolipids and also it exerted stronger therapeutic efficacy against cancer cells when incorporated into liposomal membrane comparing micellar formulation [37].

### 3.3. Polymer-Modified and Peptides-Modified Thermo-Sensitive Liposomes

Thermo-sensitive polymers present a low critical solution temperature (LCST) that corresponds to their sharp coil-to-globule transition and phase separation. These polymers are water-soluble below their cloud point (CP) while, above it, hydrogen bonds between water molecules and the polymer hydrogen bond-forming groups become weaker, resulting in less hydrated polymer chains [66]. Consequently, the polymers undergo a coil-to-globule transition, causing their precipitation. The presence of a thermo-sensitive polymer in the vesicles bilayer leads to a destruction of the membrane and to the release of the drug above LCST [67]. Thermo-sensitive polymers can be incorporated into liposomes by coupling to a hydrophobic moiety that is able to dissolve in the liposomal bilayer [68]. Since temperature promotes changes in solubility, turning polymers from hydrophilic to hydrophobic molecules, this parameter may be used to control the stabilization and destabilization of PTSL, allowing control also of the drug release and interaction with cells and serum proteins [66,69].

This strategy has been investigated in depth and one of the first thermo-sensitive polymers incorporated into liposomal membrane was the poly(*N*-isopropylacrylammide) (pNIPAM) [70]. The main drawback of this polymer is that it possesses a LCST around 32 °C in aqueous solution, which does not coincide with the basic requirement of an ideal pharmaceutical thermo-sensitive system [71]. Researchers have tried to overcome this problem through its copolymerization with appropriate co-monomers, in order to bring the LCST to a desired temperature range [70].

Even pH-sensitive monomers such as PAA may also be co-polymerized with NIPAM, conferring relevant targeting ability to different physiological districts [67]. Poly(NIPAM-*co*-propylacrylic acid) has been synthesized by reversible addition-fragmentation chain transfer giving products with a LCST of 42 °C (at pH 6.5). PTSL prepared with this polymer has been found to release 100% of loaded DOX at 42 °C within 5 min (95% of the content in 10 s at 42 °C), with a minimum release (less than 20%) at 37 °C within 30 min in serum [72].

DPPC liposomes modified with 5% of p(NIPAAm-*co*-PAA) are stable in serum and are able to release 70% and 100% of loaded DOX already after 5 min of heating at 40 °C and 42 °C, respectively. Additionally, the release from p(NIPAAm-*co*-PAA)-modified liposomes is higher at acid condition (typical of tumour cells) because of the pKa PAA (pKa = 6.7). At acid pH, in fact, the carboxyl group of PAA is protonated and promotes its coil-to-globule phase transition and consequently drug release [67].

Liposomes modified with co-polymers of NIPAM and acryloylpyrrolidine (pAPr) were already proposed by Kono and collaborators in 1999 [73]. 

Also, acrylamide (AAM) was used to modify the LCST of pNIPAM, as reported by Han and collaborators in 2006. Authors synthetized various co-polymers of NIPAM and AAM by changing the monomers ratio and observed that the variation of LCST depended on the acrylamide content [74]. Its incorporation increased the hydrophobicity of the polymer chains, resulting in the increase of LCST. Furthermore, the authors incorporated this copolymer into the bilayer of different TSL and observed that DPPC/HSPC/CHOL/DSPE-PEG_2000_-PNIPAM-AAM_17_ formulation gave the highest DOX release rates in serum. These results highlighted that the increase in hydrophobicity of the polymer induces a stronger interaction with lipid membrane and consequently enhances the drug release from liposomes. Conversely, the presence of PEG on the liposomes surface decreases their interaction with serum proteins in the bloodstream and prolongs the in vivo stability of carriers compared with polymer unmodified liposomes [74]. 

Another polymer used for the polymer-modified TSL preparation has been poly[2-(2-ethoxy) ethoxyethyl vinyl ether] (p-EOEOVE). This polymer exhibits a LCST around 40 °C, undergoing a sharply structural transition from a highly hydrophilic coil to a hydrophobic globule [75]. These PTSL are stable and retain the drug inside them below physiological temperatures. However, they exhibit a higher release of the encapsulated DOX above 40 °C reaching complete emptying within 1 min at 45 °C. Additionally, the copolymer-modified liposomes exhibit long circulating properties and biodistribution like that obtained by traditional PEG-modified liposomes [75].

Considering polymer EOEOVE potentiality in drug delivery, similar copolymers have been prepared. In particular, poly [2-(2-ethoxy) ethoxyethyl vinyl ether-block-octadecyl vinyl ether (p-EOEOVE-block-ODVE)] with a LCST around 40 °C has been synthesized: the poly(EOEOVE) block acts as the temperature-sensitive moiety, while the poly(ODVE) block behaves as anchor units [76]. In 2011, the performance of PTSL in terms of visualization and efficacy against a tumour was investigated by Katagiri et al. [77] In this study the authors incorporated hydrophobized Fe_3_O_4_ nanoparticles into the poly(EOEOVE-bODVE) liposomal bilayer via hydrophobic interactions. An alternating magnetic field was used to heat the Fe_3_O_4_ nanoparticles and to induce the release of a fluorescent marker.

The potential of the same carrier as “theranostic” nanodevice has been successful investigated by Kokuryo et al. preparing PTSL loaded with an anticancer drug, a MRI contrast agent, and a fluorescent dye with the aim of monitoring drug delivery in cancer therapy [78].

The drug release rate from grafted-polymer-TSL depends on several variables as the polymer molecular weight and grafted density. Recently, van Elk and collaborators developed *N*-(2-hydroxypropyl) methacrylamide mono/dilactate (pHPMAlac)-grafted liposomes, varying polymer molecular weights, composition and anchoring it to cholesterol [68]. Results have shown that release starts at the polymer CP temperature, and that it increases with the decrease of CH-pHPMAlac molecular weight. Furthermore, the presence of CH contributes to reducing the polymer thermo-sensitivity. The release percentage of DOX from CH-pHMAlac-grafted liposomes, with a CP at 11.5 °C, is been about 89% at 42 °C after 5 min, while that obtained with the sample possessing a CP at 25 °C has reached the same value only at 52 °C. Moreover, authors have observed that the grafting density affects the liposomes thermo-sensitivity: only 5% polymer-grafting density CH-pHMAlac ensures a fast release at the desired temperature [68].

An innovative approach to prolong the plasma half-life of TSL relies on the incorporation onto the bilayer of a thermally responsive elastin-like polypeptide (ELP), consisting of a Val-Pro-Gly-Val-Gly units [79]. ELP is reported to act like a LCST polymer, swelling below the transition temperature, due to a hydrogen bonding interaction between ELP and water molecules, and drying out above LCST, due to hydrophobic interaction, making the bilayer less flexible [80]. In 2013, Park and collaborators [72] have used modified ELP to confer thermo-sensitivity to liposomes, producing a more rigid membrane at physiological temperatures and reducing vesicle instability during blood circulation. The ELP N-terminal portion has been conjugated with a single stearyl group (C18) for anchoring to the lipid bilayer of DPPC/DSPE-PEG_2000_/CH liposomes. The resulting formulation has shown a high stability at physiological conditions and has given a significant release of encapsulated DOX after mild heating. The authors have demonstrated that ELP-liposomes can release more than 95% of drug content in 10 s at 42 °C, while less than 20% is released within 30 min at 37 °C in serum. The designed formulation has shown a plasma half-life of 2.03 h compared to a half-life of 0.92 h reported for LTSL. A significant delay in tumour growth has been achieved by ELP-liposomes in combination with high intensity focused ultrasounds compared to LTSL, already after one intravenous injection [72].

### 3.4. Multifunctional Thermo-Sensitive Liposomes (MTSL)

In the field of drug-delivery systems, the use of a single strategy is not generally sufficient to enhance anticancer therapy efficacy. Therefore, the design and development of carriers that combine different strategies in the same system, making them clinically more efficacious, are needed [81]. The thermal triggering approach has been very successful when combined with active or passive targeting strategies to further enhance its efficacy.

A synergistic combination is one that joins tumour vasculature targeting with temperature-triggered release. This is achieved by coating the thermo-sensitive vesicle surface with antigens expressed on angiogenic tumour vasculature, such as ligands for integrins vascular endothelian growth factor receptor (VEGFR), platelet-derived growth factor receptor (PDGFR), and CD13/aminopeptidase N [82].

A novel cyclic Asn-Gly-Arg (NGR) peptide not containing a disulfide bridge was synthesized by Negussie and collaborators and used to confer target properties to LSTS. In vitro fluorescence microscopy evaluation demonstrated that the peptide was actively taken up by a CD13^+^ cancer cell, showing minimal binding to CD13^−^ cells, and displayed 3,6-fold greater affinity than a linear form also when conjugated on the liposomal surface of a LSTL [22].

Kim and collaborators improved the performance of thermo-sensitive liposomes by coupling them with a cyclic arginine-glycine-aspartic acid (cRGD) peptide, able to enhance tumour accumulation through the targeting to α_v_β_3_ integrin, overexpressed in tumour vasculature and in several malignant tumours. The cellular uptake of these multifunctional vesicles was 7-fold higher than that obtained with no-targeted liposomes. These results were also confirmed by in vivo tumour accumulation experiments [83].

The use of cationic lipids and temperature-triggered release represents another strategic combination for use in cancer treatment. Cationic nanocarriers have been reported to deliver selectively anticancer drugs to cancer cells thanks to the electrostatic interactions with overexpressed anionic glycoproteins, phospholipids and proteoglycans on the tumour vasculature [84,85]. Additionally, the vesicle accumulation at the cancer site is facilitated by the irregular and slow blood flow and hyperthermia, which promotes carrier extravasion [84].

Recently, as mentioned, Dicheva and collaborators [47] have designed PEGylated cationic thermo-sensitive liposomes (CSTL) made of DPPC, DSPC, DSPE-PEG_2000_ and the cationic lipid DPTAP. These vesicles have displayed a high stability at physiological temperature and release kinetics of CF similar to that achieved by non-cationic thermo-sensitive liposomes (NCTSL). The levels of binding of CSTL to BML (benign metastatic Leiomyoma) and HUVEC (human umbilical vein endothelial cells) are higher at 37 °C compared to NCTSL. Upon binding due to their small size, CTSL are easily internalized into cancer cell and the release of CF is triggered by temperature increase and might occur both extracellularly and intracellularly, improving the therapeutic outcome. The same research group has demonstrated the effectiveness of this system by encapsulating DOX and the resulting effective mild hyperthermia-triggered drug release has confirmed the success of the dual targeting approach [46]. Additionally, the cytotoxicity of DOX-CTSL is higher than that obtained when loaded in TSL after treatment on several tumour cells lines. Furthermore, the decrease of cationic lipid in the bilayer from 10 to 7.5 mol % does not influence the cellular CTSL tumour targeting and related induction of cytotoxicity [47]. A new multifunctional formulation is also developed for the specific delivery of small interfering RNA (siRNA). To do this, Yang et al. in 2016 combined two main strategies into a single carrier to give rise to smart multifunctional TSL. The first involves direct conjugation of cell penetrating peptide (CPP) to siRNA via disulphide bonds (designated as siRNA-CPPs) which determines the carrier sensitivity to glutathione. In the second strategy, the siRNA-CPPs were encapsulated, to overcome their limitation in vivo, in TSL containing an Asparagine-Glycine-Arginine (NGR) peptide with vasculature target function. Under dual stimulus of hyperthermia and intracellular redox environment, the siRNS-CPPs/NGR-TSL has higher in vivo tumour efficacy and gene silencing efficiency rather than the free siRNA-CPPs under hyperthermia [86].

### 3.5. Thermo-Sensitive Niosomes (TSN)

Niosomes are non-ionic surfactant vesicles obtained on hydration of manufactured non-ionic surfactants, with or without joining of cholesterol or other lipids. They are vesicular systems like liposomes that can be utilized as carriers of amphiphilic and lipophilic drugs. Niosomes are promising vehicles for drug delivery and, being non-ionic, they are less dangerous and enhances the therapeutic index of the drug.

For the first time, in 2016, Tavano et al. have decided to investigate the natural characteristic of certain surfactants to transfer their thermo-sensitivity properties to niosomes. Pluronic L64 belonging to the class of poly (ethylene oxide)-poly (propylene oxide)-poly (ethylene oxide) blocks surfactants and its derivative were used to form thermo-sensitive vesicles either in the presence or absence of cholesterol, evaluating the effect of small changes in composition on the thermo-sensibility of the carriers [87]. The use of L64 as an amphiphilic constituent, with claimed stealth and thermo-sensitive functionality, may give the possibility to bypass the use of additional excipients, increasing the system biocompatibility. The in vitro calcein release studies have been performed at 25, 37 and 42 °C, that are representative of storage, physiological conditions and mild hyperthermia, showing a more pronounced calcein release from L64-based niosomes at 42 °C. Due to these promising results, authors have validated the thermo-sensitivity of these by encapsulating 5-FU in the aqueous core. Also, 5-FU release has exhibited a temperature dependence, with a marked increase at 42 °C, confirming that the temperature-sensitivity of these niosomes depends only on the surfactant characteristics and it is not affected by the chemical nature of the drug [87].

### 3.6. Thermo-Sensitive Polymersomes (TSP)

Polymersomes (Ps), also known as polymeric vesicles, are based on the self-assembly of synthetic amphiphilic branch, graft and dendritic copolymers [88]. Compared to liposomes, Ps present countless advantages such a high stability, multi-drug loading capacity and membrane property versatility, due to the great variability of the starting materials [89].

Recently, the development of stimuli-responsive polymersomes to further control the release of drugs has attracted a lot of interest and is achieved through the use of thermo-sensitive amphiphilic polymer. Already in 2006, Li and collaborators prepared polymersomes from poly[*N*-(3-aminopropyl)-methacrylamide hydrochloride]-*b*-poly(*N*-isopropylacrylamide) (PAMPA-PNIPAAM): when temperature is raised above the LCST of the PNIPAAM chains, the polymer become insoluble in water and its monomers self-assembled into the vesicular structure [90]. In the same year, Qin and collaborators studied the influence of temperature on assembly and disassembly of polymersomes encapsulating DOX, obtained from PEG-*b*-PNIPAAm with 7–20% wt PEG content [91]. By increasing the temperature, polymer and monomers gave vesicles, easily destroyable by cooling because of the increased hydrophilic character. Unfortunately, this system required very high temperatures to form polymersomes and a reduction often below the 37 °C to promote drug release; thus, it could not be effectively used in combination with mild hyperthermia treatment. To by-pass this limitation and to encourage the formation of polymersomes at room temperature, mixtures of thermo-sensitive and hydrophobic polymer blocks have been proposed. Zhou and collaborators synthesized novel polymersomes based on star copolymers presenting a hydrophobic hyperbranched poly[3-ethyl-3-(hydroxymethyl)oxetane] (HBPO) core and several hydrophilic PEG arms [92]. Unlike classical thermo-sensitive Ps, these novel carriers have been formed at room temperature and destabilized above the LCST, enhancing membrane permeability and drug release. In 2015, Liu and collaborators, designed polymersomes from hydrophilic poly(*N*-vinylcaprolactam) (PVCL) attached to a long hydrophobic PDMS poly(dimethylsiloxane) core block, generating a thermo-responsive bola amphiphile, conducting an in depth and extensive study [93]. Several poly(*N*-vinylcaprolactam)*n*-poly(dimethylsiloxane) 65-poly(*N*-vinylcaprolactam)*n*(PVCL_n_-PDMS_65_-PVCL_n_) copolymers have been synthesized with varying PVCL amounts, but only samples with a PVCL ratio between 0.36 and 0.52 are able to form stable vesicles at room temperature. Polymersomes size decreases with the temperature rise, dependently on the PVCL chain length. In fact, when the temperature increases from 25 °C to 55 °C, vesicle volume lowers with increasing PVCL length (from PVCL_10_ to PVCL_15_). Conversely, PVCL_19_-PDMS_65_-PVCL_19_ shows an increase of hydrodynamic diameter from 300 to 800 nm. Furthermore, the authors have investigated the DOX release from PVCL_10_-PDMS_65_-PVCL_10_, PVCL_15_-PDMS_65_-PVCL_15_, and PVCL_19_-PDMS_65_-PVCL_19_ based formulations both at 25 and 42 °C, in a fluid-simulating tumour environment, achieving released drug percentages of 86%, 29%, and 11% at 42 °C, respectively, and demonstrating a strong dependence on PVLC length [85]. After temperature increase, collapse and aggregation of PVLC block occurs, leading to a decrease of Ps size and to an improvement of membrane permeability, favouring the release of DOX. Also, hydration and dehydration of PVLC blocks linked to the hydrophobic PDMS block play an important role, since it is assumed that hydrophilic molecules may overcome the hydrophobic PDMS layers by the presence of transient pores at high temperature. Finally, the cytotoxicity of DOX-loaded PVCL_10_-PDMS_65_-PVCL_10_ polymersomes has been evaluated on human alveolar adenocarcinoma A549 cell line, with the result that cell viability decreased from 85% to 59% and from 71% to 50% for polymersomes containing 0.1 and 0.5 μg mL^−1^ of drug, respectively. Therapeutic efficacy stopped after 48 h for 0.1 and 0.5 μg mL^−1^ DOX-loaded vesicles, while for vesicles containing 1 and 5 μg mL^−1^ of DOX it continued until 72 h, achieving cell cytotoxicity higher than 75% and 97%, respectively [93].

## 4. Conclusions

In the field of temperature-sensitive drug delivery systems, thermo-sensitive vesicles in combination with local hyperthermia represent a powerful tool for tumour specific drug delivery. This review has shown the considerable progress in the development of thermo-sensitive vesicle formulations for targeted cancer therapy developed since 1978. Mild hyperthermia exposure has been proved to be an ideal external stimulus able to trigger localized drug release. Therefore, the combination of this strategy with a carrier sensitive to temperature changes between 39 °C and 42 °C resulted in a promising strategy to improve therapeutic efficacy. The latest studies suggest that such a carrier could be able to release rapidly and extensively a hydrophilic drug when the temperature increases a few degrees above physiological temperature.

The efficacy of TSV depends both on the specific vesicular formulation, on the encapsulated drug, and on the specific heating modality. The progress obtained in the last few years on the new formulations to improve the TSV efficacy are reported in this review, but we recommend reading the recent review about the new heating modality [94].

One limitation of many current TSV formulations is the still relatively short plasma half-life, which limits the duration available for delivery, reduces the quantity of encapsulated drug available for release, and also negatively impacts systemic toxicities. Promising results are obtained by the synergistic effect due to the combination of several approaches in the same nanodevices. We are sure that the rational design of a multifunctional thermo-responsive system has remarkable potential in target cancer therapy.

In summary, TSV represents a highly promising drug-delivery approach that has the potential for considerable clinical impact in the near future.

## Figures and Tables

**Table 1:** Most used lipids in TSL preparation.

Composition	Drug	Ref
DPPC/CH/DSPE-PEG2000)/DSPE-PEG(2000)-Folate	DOX	[31]
DPPC/HSPC/CH/DPPE-PEG_2000_/DOTA-DSPE	DOX + GD	[32]
DPPC/DSPC/DPPGOGDPPC/P-lyso-PC/DSPE-PEG_2000_	DOX	[33]
DPPC/P-lyso-PC/DSPE-PEG_2000_	DOX	[34,35]
DPPC/HSPC/CH/DSPE-PEG_2000_	DOX	[26]
DPPC/DSPC/DSPE-PEG_2000_55:40:580:15:5	DOX	[36]
DPPC/DSPCDPPC/DSPC/DPPGOG	CF	[37]
DPPC:MSPC:DSPG:DSPE-mPEG200082:8:10:4	Epirubicin	[38]
DPPC:MSPC:DSPE-PEG2000:DSPG83:3:10:4	Paclitaxel	[39]
DPPC:DSPE:PEG2000:EPC:MSPC82:11:4:3:4	Docetaxel	[40]
DPPC:CHO:DSPE-PEG90:5:5	5-Fluorouracil	[41]
DPPC:MPPC:DSPE-PEG200086:5:4	Vinorelbine	[42]
DPPC: DSPE-PEG2000: MSPC75:17:8	DOX + Vincristine	[43]
DPPC:MSPC:DSPE-PEG200085.3:9.7:5.0	DOX + ProHance^®^	[44]

## Abbreviations

AAM	acrylamide
C	Calcein
CF	carboxyfluorescein
CH	cholesterol
CP	cloud point
CPP	cell penetrating peptide
CTSL	cationic thermo-sensitive liposomes
DDS	drug delivery systems
DOX	Doxorubicin
DPPC	1,2-dypalmitoyl-*sn*-glycero-3-phosphocoline
DPPG_2_	1,2-dipalmitoyl-*sn*-glycero-3-phosphoglyceroglycerol
DPPGOG	1,2-dipalmitoyl-*sn*-glycero-3-phosphoglyceroglycerol
DSPC	distearoyl phosphocholine
DSPE-PEG_2000_	1,2-distearoyl-*sn*-glycero-3-phosphoethanolamine-*N*-methoxy(PEG)-2000
ELP	elastine-like polypeptide
FCS	fetal calf serum
GD	Gadolinium
Gem	Gemcitabine
HBPO	hydrophobic hyperbranched poly[3-ethyl-3-(hydroxymethyl)oxetane]
HePC	hexadecylphosphocholine
HAS	human serum albumin
HSPC	hydrogenated soyphosphocholine
IgG	immunoglobulin type G
LCST	low critical solution temperature
LP	Lysolipids
LTSL	lysolipids thermo-sensitive liposomes
LTST	low thermo-sensitive liposomes
MPPC	Monopalmitoylphosphatidylcholine
MSPC	monostearoylphosphatidylcholine
MTSL	multifunctional thermo-sensitive liposomes
NCTSL	non-cationic thermo-sensitive liposomes
PAA	propyl acrylic acid
PAMPA-PNIPAAM	poly[*n*-(3-aminopropyl)-methacrylamidehydrochloride]-*b*-poly(*n*-isopropylacrylamide)
PDGFR	patelet-derived growth factor receptor
PDMS	poly(dimethylsiloxane)
pEOEOVE	poly[2-(2-ethoxy)ethoxyethyl vinyl ether]
pEOEOVE-block-ODVE	poly [2-(2-ethoxy)ethoxyethyl vinyl ether-block-octadecyl vinyl ether] EOEOVE-block-ODVE
pHPMAlac	*N*-(2-hydroxypropyl)methacrylamide mono/dilactate
P-Lyso-PC	1-palmitoyl-2-hydroxy-*sn*-glycero-3-phosphocholine
pNIPAM	poly(*N*-isopropylacrylammide)
PpTSL	peptides thermo-sensitive liposomes
Ps	polymersomes
PTSL	polymer thermo-sensitive liposomes
PVCL	poly(*N*-vinylcaprolactam)
PVCL_n_-PDMS_65_-PVCL_n_	poly(*N*-vinylcaprolactam)*n*-poly(dimethylsiloxane)65-poly(*N*-vinylcaprolactam)n
RES	reticulo-endothelial system
*T*_m_	phase transition temperature
siRNA	small interfering RNA
TSL	thermo-sensitive liposomes
TSN	thermo-sensitive niosomes
TSP	thermo-sensitive polymersomes
TSV	temperature-sensitive vesicles
VEGFR	endothelian growth factor receptor
